# Safety assessment of fermented jackfruit (*Artocarpus heterophyllus*) pulp and leaves in Sprague‐Dawley rats

**DOI:** 10.1002/fsn3.1734

**Published:** 2020-07-01

**Authors:** Sarah Sabidi, Soo Peng Koh, Shazwan Abd Shukor, Shaiful Adzni Sharifudin, Yun Shin Sew

**Affiliations:** ^1^ Food Science and Technology Research Centre MARDI Headquarters Persiaran MARDI‐UPM Serdang Malaysia; ^2^ Biotechnology & Nanotechnology Research Centre MARDI Headquarters Persiaran MARDI‐UPM Serdang Malaysia

**Keywords:** fermentation, jackfruit leaves, jackfruit pulp, Sprague‐Dawley rats, toxicity

## Abstract

Fermented jackfruit (*Artocarpus heterophyllus*) extracts were produced using pure symbiotic culture of bacteria and yeast (SCOBY) under controlled fermentation process. Both female and male Sprague‐Dawley rats were orally administrated with 4,000 mg/kg of fermented jackfruit pulp and leaves extracts for 28 consecutive days. Body weight of rats was recorded at 1‐week interval until necropsy day. There was no mortality reported along the experiment with no significant differences (*p* > .05) record among organ histopathology and blood biochemical parameters in treated groups when compared to control group. Interestingly, there were significant differences (*p* < .05) in the lower body weight gained of treated rats groups as opposed to control group, indicating the potential anti‐obesity effect of fermented jackfruit extracts. In conclusion, no toxicity symptoms were observed in 28 days oral administration toxicity study of fermented jackfruit pulp and leaves extracts in Sprague‐Dawley rats for both sexes.

## Graphical abstract

No toxicity symptoms were observed in Sprague‐Dawley rats for both sexes after 28 days oral administration with fermented jackfruit pulp and leaves. There were significant differences (*p* < .05) of lower body weight gained observed in the treated rat groups as opposed to control group, indicating the potential anti‐obesity effect of fermented jackfruit products.

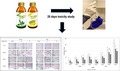

## 1 INTRODUCTION

The increase of health awareness due to change of eating habits and living standards has shifted consumer preference into nutritious, healthy, and disease preventive food which promises wider health benefits. This phenomenon has changed the primary role of food as main energy source to a new concept of biologically active food components for maintaining good health (Granato, Branco, Nazzaro, Cruz, & Faria, 2010). The presence of various phytochemicals, antioxidant, vitamin, mineral, and dietary fiber contents confer fruits as an ideal functional food. Unfortunately, the short shelf life of fruits needs proper processing techniques in order to maintain their nutritious market value (Panghal et al., 2017).

Jackfruit (*Artocarpus heterophyllus*) has been widely accepted by consumers as highly nutritious fruits and a desirable fruit crop to be grown due to its rich bioactive compounds (Swami, Thakor, Haldankar, & Kalse, 2012). Past findings have reported that jackfruit is rich in phenolic compounds which contributing to its multiple pharmacological properties for the treatment of antidiabetic, inflammatory, wound healing, and fungal infection. (Baliga, Shivashankara, Haniadka, Dsouza, & Bhat, 2011; Biworo, Tanjung, Iskandar, Khairina, & Suhartono, 2015; Jagtap & Bapat, 2010). Besides possessing a unique flavor, jackfruits also rich in phytochemical compounds serve as vital components for various health‐promoting benefits (Jagtap, Panaskar, & Bapat, 2010). Currently, jackfruit pulp is processed to make chips, chutneys, jellies, candies, or added to favor food products like ice cream, beverages, or desserts. However, overproduction of jackfruits particularly during harvest season and its short shelf life might cause economic lose to farmers. The short shelf life of jackfruits can be improved by manipulating high sugar content of the fruit pulp which is a potential substrate for fermentation process (Kumoro, Sari, Pinandita, Retnowati, & Budiyati, 2012). On the other hand, jackfruit leaves are commonly used as a traditional folk medicine for maintaining good health. However, its awful taste is still unfavorable by consumers. Therefore, abundant of jackfruit leaves are disposed as agricultural waste due to lack of product development study. Past toxicological evaluation had reported no toxicity symptoms observed in Albino mice either fed with methanolic or aqueous extract of jackfruit leaves on a dose of 2,000 mg/kg (Bhattacharjee & Dutta, 2013).

Microbial fermentation is simple, natural yet powerful techniques to preserve food and improve the biological functionality of raw materials with the aid of microbial works. Fermented foods are more palatable and enriched with aroma and taste, as a consequence of the bio‐fermentation process that capable to hydrolyze macronutrient molecular into easily digestible phytonutrients as being reported in many fermented foods (Kang, Yang, Dominy, & Lee, 2010; Marotta, Celep, Cabeca, & Polimeni, 2012). In Malaysian Agricultural Research and Development Institute (MARDI), we have developed a new bio‐fermentation process to produce fermented jackfruit pulp (JP) and leaves (JL) extracts with improved functional bioactivities using pure symbiotic culture of bacteria and yeast (SCOBY) from MARDI's Collection of Functional Food Cultures (Koh et al., 2018). The traditional fermented sugared black tea (Kombucha tea) was produced using symbiotic aggregate of various yeast and bacteria. Even though this fermented tea has been claimed as healthy elixir with medicinal properties, however, there is limited evidence available which raise safety concern that it may cause health risk. Hyperthermia, hepatic dysfunction, lactic acidosis, respiratory failure, or fatal are few toxicity cases had been reported upon Kombucha tea ingestion (Kole, Jones, Christensen, & Gladstein, 2009). Our SCOBY jackfruit extracts taste acidic due to the presence of multiple organic acids produced as a result of microbiological activities as shown in Table 1. To date, there is no safety assessment carried out for this newly developed fermented JP and JL extracts. Because of this reason, we were focused on the safety aspect evaluation of fermented jackfruit JP and JL extracts in rats fed continuously at a high dosage of 4,000 mg/kg. The body weight, hematological, biochemical, and histopathological parameters were analyzed as recommended in the Nurul et al.'s study (2018). The main objective of this experiment was to determine the possible toxic effect of fermented JP and JL extracts on both female and male Sprague‐Dawley rats after oral administration for 28 consecutive days.

**TABLE 1:** The pH, brix value and organic acids profile of fermented jackfruit extracts

Sample	pH value	Brix value	Acetic acid	Citric acid	Oxalic acid (µg/ml)	Malic acid	Kojic acid	Quinic acid	Succinic acid
JL	3.15 ± 0.02	8.23 ± 0.05	16,892.80 ± 1,269.00	276.05 ± 24.74	16.38 ± 0.27	86.58 ± 4.99	7.21 ± 0.43	478.88 ± 20.23	0.00 ± 0.00
JP	3.06 ± 0.01	11.18 ± 0.04	16,012.58 ± 3,576.00	871.63 ± 14.04	17.33 ± 0.74	84.92 ± 8.24	11.04 ± 0.41	201.21 ± 6.29	84.09 ± 4.56

Note

Each value in Table represents the mean ± standard deviation from triplicate sample analyses

Abbreviations: JL, fermented jackfruit leaves; JP, fermented jackfruit pulp.

## 2 MATERIALS AND METHODS

### 2.1 Preparation of fermented jackfruit extracts

The jackfruit (*Artocarpus heterophyllus* L.) with variety of Tekam Yellow J33 was purchased from a local jackfruit plantation in Lanchang, Pahang. The ripened jackfruits pulp was separated from the seeds and thoroughly cleaned with filtered water before minced into smaller chunks and oven‐dried (45°C) to produce pulp granule. The jackfruit leaves need to wash thoroughly to remove all dirts before oven‐dried to make leaves powder. Both dried substrates were packed in the aluminum bag and stored in a chiller (4°C) for future use.

Both jackfruit leaves and pulp suspension at the concentration of 5% (w/v) were prepared as a growth medium for the production of fermented jackfruit pulp (JP) and leaves (JL) extracts, respectively. Both jackfruit pulp and leaves suspension were inoculated with two types of microorganisms: (a) yeast (*Dekkera* sp.) and (b) acetic acid bacteria (*Komagataiebacter* sp.) were selected from MARDI's Collection of Functional Food Cultures. After a week of fermentation process, the supernatant was collected after centrifuged at 11,200 *g* for 10 min to remove microbes and substrate residue. The extracts were named as fermented JP and JL, which were produced from the substrate of jackfruit pulp and leaves, respectively.

### 2.2 Organic acids analysis

Analyses of organic acids profile of fermented jackfruit pulp and leaves extracts were carried out with a high‐performance liquid chromatography (HPLC), Alliance Separation Module (Waters, 2695), equip with a diode array detector (Waters, 2996) as described in Koh et al.'s study (2019). Quantification of organic acids was determined from a calibration curve obtained by injecting known amount of each organic acid as external standard. All analyses were conducted in triplicate.

### 2.3 Ultra high‐performance liquid chromatography (UHPLC)—quadrupole time‐of‐flight (QTOF) mass spectrometry analysis

The UHPLC system was performed on ACQUITY UPLC I‐Class system, consisting of a binary pump, a vacuum degasser, an auto‐sampler, and a column oven and was coupled to a Vion IMS QTOF hybrid mass spectrometer from Waters, equipped with a lock spray ion source. The compounds of fermented jackfruit extracts were chromatographically separated using a column ACQUITY UPLC HSS T3 (100 mm × 2.1 mm × 1.8 μm) and with column oven maintained at 40°C. A linear binary gradient of water with 0.1% formic acid and acetonitrile was used as mobile phase A and B, respectively. The mobile system was run according to gradient profile as follows: 0 min, 1% B; 0.5 min, 1% B; 16.00 min, 35% B; 18.00 min, 100% B; 20.00 min, 1% B. The flow rate was set to 0.6 ml/min and the injection volume was 1 μl. The ion source was operated in negative electrospray ionization (ESI) mode under the following specific conditions: capillary voltage, 1.50 kV; reference capillary voltage, 3.00 kV; source temperature, 120°C; desolvation gas temperature, 550°C; desolvation gas flow, 800 L/hr, and cone gas flow, 50 L/hr. Nitrogen (>99.5%) was employed as desolvation and cone gas. Data were acquired in high‐definition MSE (HDMSE) mode in the range m/z 50–1,500 at 0.1 s/scan. The scan with different collision energies (CE) was acquired during the run: a low‐energy (LE) scan at a fixed CE of 4 eV, and a high‐energy (HE) scan where the CE was ramped from 10 to 40 eV. Argon (99.999%) was used as collision‐induced dissociation (CID) gas.

### 2.4 Animals

Six weeks old of male and female Sprague‐Dawley rats were purchased from A‐Sapphire Enterprise (001303794‐M) which supplied healthy animal for laboratory experiment. All rats were placed in the Animal Metabolism, Toxicology and Reproductive Centre (MARDI). The rats were maintained under standard condition at 25 ± 2°C with 12 hr light/dark cycles. All animals were acclimatized for 2 weeks in cage, provided with standard sawdust bedding, distilled water, and commercial pellet Gold Coin, Malaysia (crude protein 21%, crude fiber 5%, crude fat 3%, moisture 13%, ash 8%, calcium 0.8%, and phosphorus 0.4%). The study was conducted according to the guidelines and was approved by the Animal Ethics Committee of MARDI (20170420/R/MAEC 00008).

### 2.5 Safety assessment study

Male (*n* = 9) and female Sprague‐Dawley rats (*n* = 9) at the approximate average weight of 140 g were separated into three groups; which were normal control rats group with access of food pellet and distilled water ad libitium and another two treated rat groups were fed with 4,000 mg/kg body weight of fermented JP and JL extract, respectively. The jackfruit fermented extracts were administered orally by oral gavage technique for 28 consecutive days, and the observation of morbidity and mortality was carried out at least twice daily. The rats' body weights gained and glucose level were measured once a week. On day 29, rats were sacrificed in the CO_2_ chamber and blood was drawn afterward via brachial artery. The sacrificed rats were then dissected and the organs (stomach, liver, kidney, and spleen) were harvested and weighed.

### 2.6 General behavior and mortality

All rats were individually observed daily for abnormal behavior and appearance especially in changes in skin, fur, eyes, nose, and fecal. Changes in hyperactivity, tremors, ataxia, salivation, diarrhea, lethargy, and sleep of all rats were examined as well for 28 consecutive days.

### 2.7 Body weight and blood glucose measurement

Each body weight and blood glucose of rats was recorded at the initiation treatment and once weekly until the day of necropsy. Necropsy of all rats was carried on Day 29 and individual organ of liver, spleen, kidney, and stomach for each rat group were harvested.

### 2.8 Hematology analysis

All rats' blood samples were withdrawn from the brachial artery and stored in tubes contain anticoagulant EDTA‐2K. Red blood cell, platelets, white blood cell, and lymphocytes' count were obtained by running automated hematology analyzer (Exigo Blood Hematology Analyzer).

### 2.9 Serum biochemistry

Blood samples were collected from the brachial artery of all rats. Serum samples were obtained by centrifugation (Centrifuge S417R, Eppendorf) at 1,792 *g* for 10 min under the controlled temperature of 4°C. Then, serums samples were analyzed using a clinical chemistry autoanalyzer (DIRUI CS‐300) to identify alanine aminotransferase (ALT), alkaline phosphatase (ALP), aspartate aminotransferase (AST), total bilirubin, total protein, creatinine, urea, and cholesterol readings.

### 2.10 Histopathology examination

The liver, spleen, kidney, and stomach organ were fixed in neutral buffered 10% formalin solution. All fixed organs were embedded in paraffin, sectioned, stained with hematoxylin and eosin (H&E), and examined under microscope (Leica).

### 2.11 Statistical analysis

Results are presented as mean ± standard deviation of replicated samples. Data were subjected to one‐way analysis of variance (ANOVA) and multiple comparisons were performed by Duncan's test. Statistical significance was set at the level of *p* < .05. All analyses were performed using statistical analysis software, IBM SPSS Statistic 22.0 (IMB Corp.).

## 3 RESULTS

### 3.1 General behavior and mortality observation

Daily oral administration of 4,000 mg/kg body weight of fermented JP and JL extracts for 28 consecutive days did not induce any obvious symptom of toxicity in rats. There was an absence of hyperactivity, tremors, ataxia, salivation, diarrhea, and lethargy detected. Most importantly, no incidence of mortality and no abnormal behavior were recorded throughout the experiment.

### 3.2 Net weight gain and glucose measurement

**FIGURE 1:**
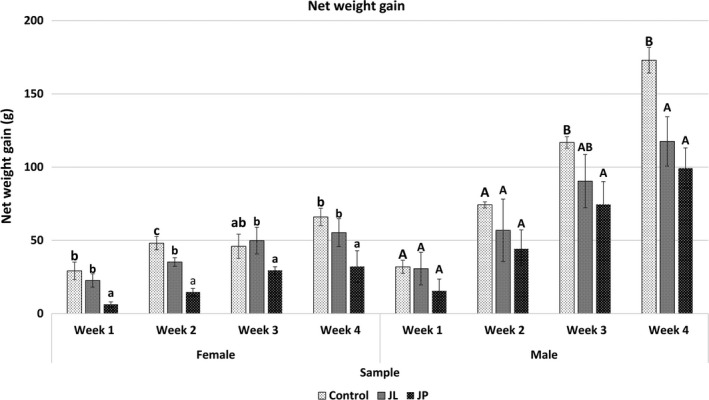
Net weight gain (g) of control and treated rats group with fermented jackfruit extracts. Means within the control and treated rats in female group with different small letter are significantly (*p* <0.05) different. Means within the control and treated rats in male group with different capital letter are significantly (*p* <0.05) different. JL, fermented jackfruit leaves; JP, fermented jackfruit pulp

**FIGURE 2:**
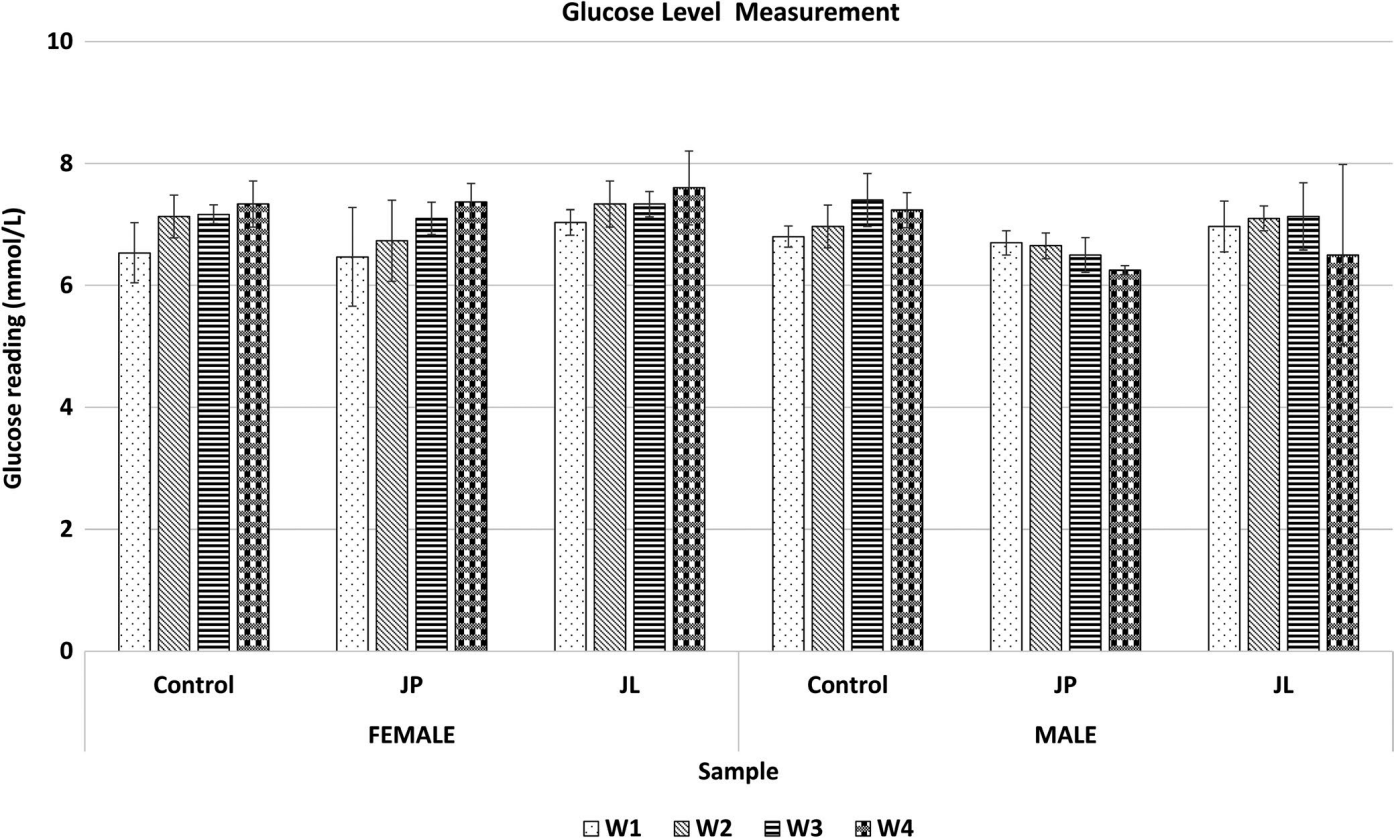
Glucose level reading (nonfasting state) of control and treated rats group with fermented jackfruit extracts. JL, fermented jackfruit leaves; JP, fermented jackfruit pulp

Body weight of both male and female rats was found gain gradually in every week. Surprisingly, both female and male treated rats (JL and JP group) showed significantly lower (*p* < .05) in weight gain over a time when compared to the control group (Figure 1). Generally, the glucose level reading (nonfasting state) showed no significant difference (*p* > .05) between control and treated rat group (Figure 2).

### 3.3 Organ weight, macroscopic and microscopic observation

**FIGURE 3:**
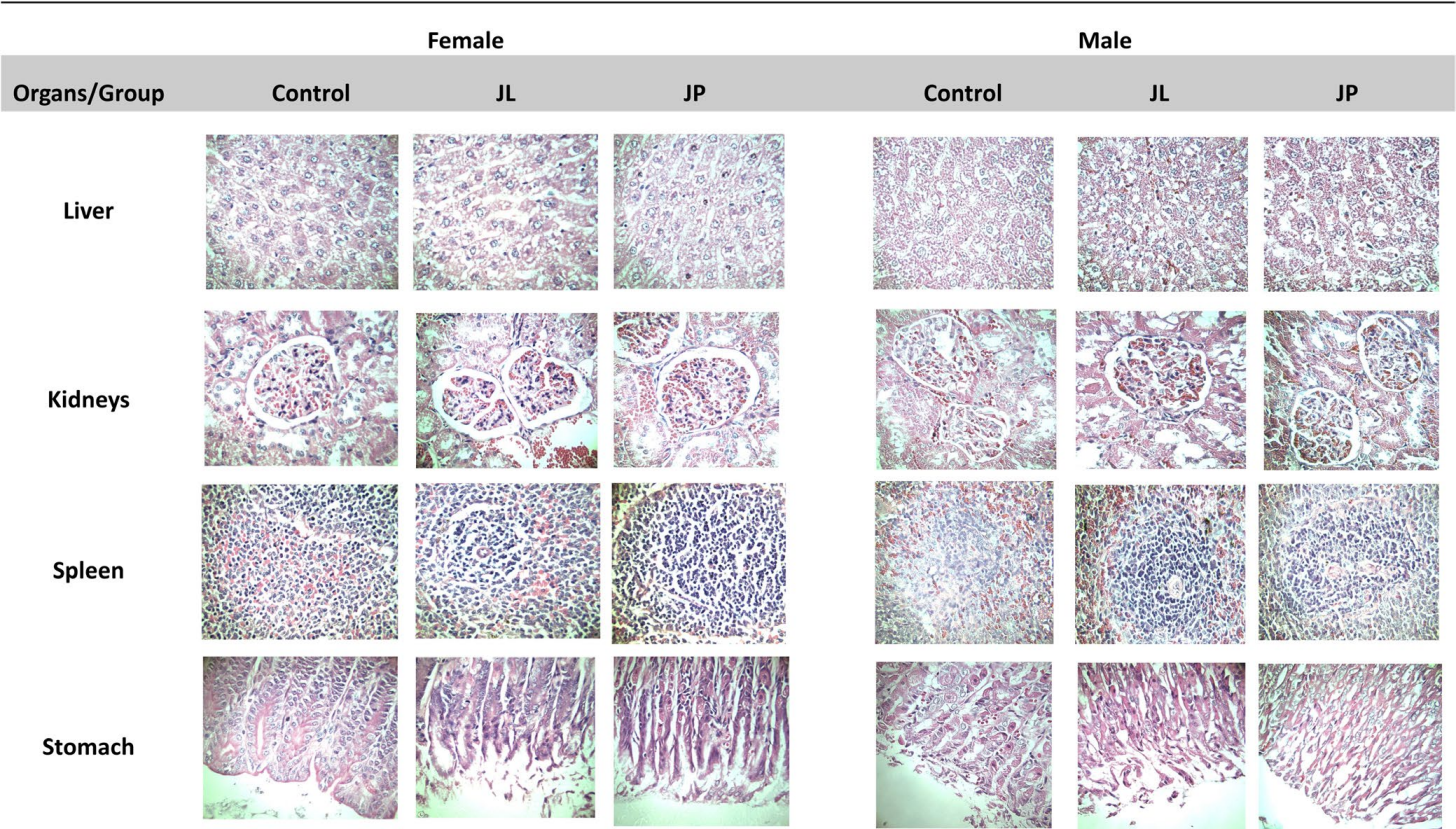
Histopathology assessment of the rat's organs (liver, kidney, spleen, and stomach) for control and treated rats group with fermented jackfruit extracts (magnification: 10×). JL, fermented jackfruit leaves; JP, fermented jackfruit pulp

The relative organ weight was calculated as (organ/body weight) × 100%. Most of the organ weight data shown no statistically significant differences (*p* > .05) were observed, except for liver for both male and female rats treated with fermented JP and JL extracts (Table 2). However, no abnormal physical appearances or swelling symptom on liver were observed. Furthermore, histopathology analysis on liver, kidney, spleen, and stomach indicated normal cells with no inflammatory symptoms (Figure 3).

**TABLE 2:** Percentage of relative organ per weight of rats after 28 days treatment with fermented jackfruit pulp (JP) and leaves (JL) extracts

Organ	Relative organ body weight (%)					
	Female			Male		
	Control	JL	JP	Control	JL	JP
Liver	4.57 ± 0.50^b^	3.79 ± 0.07^a^	3.86 ± 0.15^a^	4.75 ± 0.23^B^	4.20 ± 0.08^A^	3.94 ± 0.01^A^
Spleen	0.27 ± 0.09^a^	0.25 ± 0.04^a^	0.27 ± 0.05^a^	0.29 ± 0.02^A^	0.25 ± 0.07^A^	0.27 ± 0.04^A^
Kidney	0.73 ± 0.04^a^	0.76 ± 0.01^a^	0.80 ± 0.02^a^	0.92 ± 0.04^A^	0.84 ± 0.04^A^	0.85 ± 0.03^A^
Stomach	0.61 ± 0.02^a^	0.62 ± 0.08^a^	0.64 ± 0.06^a^	0.52 ± 0.01^B^	0.50 ± 0.01^B^	0.42 ± 0.05^A^

Note

The results of both male and female Sprague‐Dawley rats (n=6) of each group are expressed as mean±standard deviation. Means within the control and treated rats in female group with different small letter are significantly (*p* <0.05) different. Means within the control and treated rats in male group with different capital letter are significantly (*p* <0.05) different.

Abbreviations: JL, fermented jackfruit leaves; JP, fermented jackfruit pulp.

### 3.4 Hematology analysis

Results of the hematological parameters on red blood cell, white blood cell, and lymphocytes' count displayed slightly significant differences (*p* < .05) between control and both treatment (JL and JP) groups except platelets (Table 3). Nevertheless, all hematology readings were falls within the normal reference range for all hematology parameters (Lillie, Temple, & Florence, 1996; Petterino & Argentino‐Storinob, 2006).

**TABLE 3:** Hematological profile of control and treated rats with fermented jackfruit extracts

Parameter	Male			Female		
	Control	JL	JP	Control	JL	JP
Red blood cell (10^12^/L)	7.37 ± 0.07^a^	8.10 ± 0.04^ab^	8.20 ± 0.62^b^	7.56 ± 0.35^AB^	8.02 ± 0.70^A^	8.02 ± 1.00^B^
Platelets (10^9^/L)	1,071.00 ± 20.51^a^	1,195.00 ± 20.51^a^	1,128.00 ± 39.60^a^	1,031.00 ± 240.42^A^	825.00 ± 370.52^A^	1,095.00 ± 151.32^A^
White blood cell (10^9^/L)	6.20 ± 0.57^a^	5.00 ± 0.85^b^	8.35 ± 2.05^ab^	16.10 ± 0.57^AB^	13.35 ± 0.35^A^	16.30 ± 5.09^B^
Lymphocytes (10^9^/L)	3.90 ± 0.71^a^	6.20 ± 0.99^b^	3.40 ± 0.71^b^	11.40 ± 2.56^AB^	9.35 ± 0.64^A^	9.35 ± 0.64^B^

Note

The results of both male and female Sprague‐Dawley rats (n=6) of each group are expressed as mean±standard deviation. Means within the control and treated rats in female group with different small letter are significantly (*p* <0.05) different. Means within the control and treated rats in male group with different capital letter are significantly (*p* <0.05) different.

Abbreviations: JL, fermented jackfruit leaves; JP, fermented jackfruit pulp.

### 3.5 Serum biochemistry

Serum biochemistry data for both female and male rats are shown in Table 4. No significant differences (*p* > .05) were observed in serum biochemistry parameter for ALT, ALP, AST, total bilirubin, total protein, creatinine, urea, and cholesterol for all rat group except for ALT reading for male rats and cholesterol level for female rats which were treated with fermented JP and JL products, whereby it exhibited slight higher reading when compared to control group. Nevertheless, all serum biochemistry parameters were fell within the normal physiological range of Sprague‐Dawley rats ages 8–16 weeks (Petterino & Argentino‐Storinob, 2006).

**TABLE 4:** Serum biochemistry parameters of control and treated rats with fermented jackfruit extracts

Parameter	Female			Male		
	Control	JL	JP	Control	JL	JP
ALT reading (U/L)	51.00 ± 3.27^a^	56.50 ± 4.49^a^	55.50 ± 9.39^a^	53.50 ± 2.86^A^	60.50 ± 1.22^B^	70.50 ± 0.41^AB^
ALP reading (U/L)	183.00 ± 15.51^a^	201.50 ± 28.99^a^	205.50 ± 0.41^a^	269.50 ± 42.87^A^	275.00 ± 4.90^A^	326.00 ± 60.42^A^
AST reading (U/L)	139.50 ± 2.86^a^	141.50 ± 2.04^a^	142.50 ± 28.17^a^	95.00 ± 11.43^A^	122.50 ± 6.94^A^	144.50 ± 4.49^A^
Total bilirubin (mg/dl)	0.44 ± 0.01^a^	0.39 ± 0.00^a^	0.42 ± 0.00^a^	0.39 ± 0.00^A^	0.40 ± 0.00^A^	0.43 ± 0.01^A^
Total protein (g/L)	78.15 ± 0.78^a^	80.30 ± 2.20^a^	84.80 ± 2.78^a^	75.85 ± 1.18^A^	88.20 ± 2.12^A^	87.70 ± 2.94^A^
Creatinine (µmol/L)	47.00 ± 4.08^a^	50.50 ± 2.86^a^	56.50 ± 4.49^a^	49.50 ± 2.86^A^	51.50 ± 1.22^A^	54.50 ± 2.04^A^
Urea (mmol/L)	7.25 ± 0.53^a^	6.65 ± 0.37^a^	7.50 ± 0.00^a^	6.35 ± 0.20^A^	6.70 ± 0.33^A^	7.50 ± 0.33^A^
Cholesterol (mmol/L)	1.54 ± 0.22^a^	1.79 ± 0.16^b^	1.75 ± 0.26^ab^	1.19 ± 0.00^A^	1.42 ± 0.03^A^	1.44 ± 0.13^A^

Note

The results of both male and female Sprague‐Dawley rats (n=6) of each group are expressed as mean±standard deviation. Means within the control and treated rats in female group with different small letter are significantly (*p* <0.05) different. Means within the control and treated rats in male group with different capital letter are significantly (*p* <0.05) different.

Abbreviations: ALP, alkaline phosphatase; ALT, alanine aminotransferase; AST, aspartate aminotransferase; JL, fermented jackfruit leaves; JP, fermented jackfruit pulp.

## 4 DISCUSSION

In this study, two types of fermented jackfruit pulp (JP) and leaves (JL) extracts were produced using selected mixed SCOBY strains under controlled bio‐fermentation process. As expected, both fermented jackfruit extracts were found to be more palatable with improved functional benefits after gone through microbial fermentation process. Both fermented JP and JL extracts were acidic with the pH value of 3 because of the presence of multiple organic acids with the predominant content of acetic acid, citric acid, and quinic acid as shown in Table 1. Acetic acids plays important role in our body health as it contributes to the multiple biological function, particularly in reducing obesity, anti‐inflammatory effect and improving glucose tolerance in type 2 diabetic rats (Beh et al., 2017; Yamashita, 2015). However, prolonged consumption of acidic food may have adverse effect and limited evidence available could rise concern on possible serious health risk as being reported by Kole et al. (2009). Therefore, it is crucial to investigate the toxicity aspect of consuming fermented jackfruit extracts.

Throughout 28 days oral administration of fermented jackfruit JP and JL extracts at the high dose of 4,000 mg/kg, the data presented in the results have supported the safety consumption of fermented jackfruit extracts. Raza, Al‐Shabanah, El‐Hadiyah, and Al‐Majed (2002) reported that the body weight of rats might change as a consequence of adverse effect due to the presence of chemical or toxic compounds. The changes will consider as statistically significant if the current body weight loss is more than 10% from the initial body weight. However, in this study, all control and treated rats groups showed a gradually rise in total weight gained throughout 1 month toxicity study which signed a good safety response.

Surprisingly, there was a significant lower (*p* < .05) in body weight gained in both fermented jackfruit treated male and female rats observed after 1‐month treatment when compared to control group (Figure 1). This phenomenon indicated that the fermented jackfruit extracts may possess anti‐obesity effects due to the presence of acetic acid content (~1.6%), a bioactive metabolite produced from SCOBY strains fermentation process. This data was supported by the scientific findings which revealed that the presence of acetic acid content in vinegar contributing to its anti‐obesity and antiglycemic effects via increasing satiety (Beh et al., 2017; Ostman, Granfeldt, Persson, & Bjὃrck, 2005). Besides acetic acid, the presence of quinic acids in both fermented jackfruit extracts also reported to have anti‐adipogenic and lipolytic properties as described by Dungjai et al. (2018). The UHPLC‐QTOF mass spectrometry analysis has identified other potential anti‐obesity compounds presents in both fermented jackfruit extracts such as caffeic acid, apigenin, baicalin, catechin, epicatechin‐3‐gallate, galactose, kaempferol glycoside, naringin, neomangiferin, pectolinarin, quercetin, tiliroxide, and toosendanin that have been confirmed by other researchers. The presence of these compounds further supporting the potential of fermented jackfruit extracts as a new anti‐obesity therapeutic agent. Prolong consumption of fermented jackfruit extracts may change in gut microbiota, indirectly leading to lower body net weight gain and its involvement on energy homeostasis (Clarke et al., 2012). Therefore, future studies will be focused on anti‐obesity effects of fermented jackfruit extracts and its influence on the gut microbiota profile.

The nonfasting state of glucose level reading showed no significant difference (*p* > .05) between the control and both treatment groups during 28‐day oral administration (Figure 2), indicating neither hyperglycemia nor hypoglycemia effect of consuming fermented jackfruit products. The internal rats organ weight can be used as one of the relevant toxicity indicators as it could lead to morphological changes. In general, the organs weight of both female and male treated rats was comparable to control group. The study also revealed no mortality and adverse effect recorded on the rats in the treatment group as shown in the histopathology findings (Figure 3) and relative organ weight data (Table 2). In comparison to control rats group, the slightly lower liver weight observed in both treated male and female rat groups might due to the smaller rat size as a consequence of loss weight after 1 month of consuming fermented jackfruit extracts. Nevertheless, all liver, kidney, spleen, and stomach cell of both treated rats group remained in a healthy cell condition even though these rats have been subjected to prolonged consumption of high dose fermented jackfruit extracts.

Toxicity of a test material can be measured using biochemical parameters as an indicator. The serum biochemical data can be used to detect whether the fermented jackfruits extracts may affect the hematopoiesis or leukopoiesis in rats. Generally, hematological profile for both control and treated rats group were in normal reference range even though there was a significant difference recorded between treated group and control group for both gender (Table 3). Hence, fermented jackfruit extracts did not interfere with hematological profile in rat blood, supporting the evidence that it is safe to be consumed. The serum biochemistry of control and treated rats was scrutinized to investigate the possible health risk and toxicity effect of consuming fermented jackfruit extracts. As expected, there were no abnormalities observed in serum biochemistry parameters of rats treated with fermented jackfruit extracts when compared to the control group (Table 4). The normal levels of ALT, ALP, AST, total bilirubin, total protein, creatinine, urea and cholesterol are good indicators of liver and kidney functions (Hilaly, Israili, & Lyoussi, 2004). The normal serum parameters level showed that the oral administrations of high dose fermented jackfruit extracts did not change the physiology function of liver and kidneys rats. This finding was further supported by the histopathology evaluation on liver, kidney, spleen, and stomach which indicating that high dose consumption of fermented jackfruit extracts did not show adverse effect on the morphology of both male and female rat organs (Figure 3).

## 5 CONCLUSIONS

In conclusion, there was no toxicological significant symptom observed throughout the 28 days oral administration of high dose fermented JP and JL extracts in Sprague‐Dawley rats as supported by the evidence data from blood glucose, hematology, histopathology, and serum biochemistry profile of both control and treated rats groups. All data collected are found within the reference range reading in normal features of rat. The normal rat behaviors and gradually increase body weight gained throughout 28 days safety assessment study indicating no harmful side effect of consuming fermented jackfruit extracts.

## CONFLICT OF INTEREST

The authors declare that the research was conducted in the absence of any commercial or financial relationships that could be construed as a potential conflict of interest.
